# A Maternal Serum Metabolite Ratio Predicts Large for Gestational Age Infants at Term: A Prospective Cohort Study

**DOI:** 10.1210/clinem/dgab842

**Published:** 2021-11-19

**Authors:** Ulla Sovio, Neil Goulding, Nancy McBride, Emma Cook, Francesca Gaccioli, D Stephen Charnock-Jones, Deborah A Lawlor, Gordon C S Smith

**Affiliations:** 1 Department of Obstetrics and Gynaecology, University of Cambridge; NIHR Cambridge Biomedical Research Centre, Cambridge, UK; 2 Centre for Trophoblast Research, Department of Physiology, Development and Neuroscience, University of Cambridge, Cambridge, UK; 3 NIHR Bristol Biomedical Research Centre, Bristol, UK; 4 MRC Integrative Epidemiology Unit at the University of Bristol, Bristol, UK; 5 Population Health Sciences, Bristol Medical School, Bristol, UK

**Keywords:** pregnancy, metabolomics, large for gestational age, macrosomia, prediction

## Abstract

**Context:**

Excessive birth weight is associated with maternal and neonatal complications. However, ultrasonically estimated large for gestational age (LGA; >90th percentile) predicts these complications poorly.

**Objective:**

To determine whether a maternal serum metabolite ratio developed for fetal growth restriction (FGR) is predictive of birth weight across the whole range, including LGA at birth.

**Methods:**

Metabolites were measured using ultrahigh performance liquid chromatography-tandem mass spectroscopy. The 4-metabolite ratio was previously derived from an analysis of FGR cases and a random subcohort from the Pregnancy Outcome Prediction study. Here, we evaluated its relationship at 36 weeks of gestational age (wkGA) with birth weight in the subcohort (n = 281). External validation in the Born in Bradford (BiB) study (n = 2366) used the metabolite ratio at 24 to 28 wkGA.

**Results:**

The inverse of the metabolite ratio at 36 wkGA predicted LGA at term [the area under the receiver operating characteristic curve (AUROCC) = 0.82, 95% CI 0.73 to 0.91, *P *= 6.7 × 10^−5^]. The ratio was also inversely associated with birth weight z score (linear regression, beta = −0.29 SD, *P *= 2.1 × 10^−8^). Analysis in the BiB cohort confirmed that the ratio at 24 to 28 wkGA predicted LGA (AUROCC = 0.60, 95% CI 0.54 to 0.67, *P *= 8.6 × 10^−5^) and was inversely associated with birth weight z score (beta = −0.12 SD, *P *= 1.3 × 10^−9^).

**Conclusions:**

A metabolite ratio which is strongly predictive of FGR is equally predictive of LGA birth weight and is inversely associated with birth weight across the whole range.

A high absolute birth weight, independent of gestational age, is called macrosomia and is usually defined as >4000 g (1). Macrosomia is associated with an increased risk of maternal and neonatal complications; moreover, the children have a higher risk of obesity, diabetes, and cardiovascular diseases in later life (2). Macrosomia is jointly determined by increased size for gestational age [called “large for gestational age” (LGA)] and a prolonged duration of pregnancy. LGA is commonly defined as an estimated fetal weight (EFW) or an actual birth weight >90th percentile for the given gestational age. Hence, the risk of a macrosomic birth weight is increased if the fetus is LGA and more so if the pregnancy is prolonged. Consequently, 1 approach to preventing macrosomia and its associated complications is to identify LGA fetuses and to shorten the duration of pregnancy by earlier delivery. A Cochrane review of randomized trials in nondiabetic women showed that induction of labor for suspected macrosomia based on ultrasonic EFW at 37 to 40 weeks of gestational age (wkGA) does not alter the risk of cesarean delivery but reduces the risks of birth fractures and shoulder dystocia (3).

The current approach to ultrasound scanning to detect LGA in pregnancy is to target women at high risk—for example, those with diabetes mellitus or who have a symphyseal-fundal height above the upper limit of the normal range for gestational age (called “large for dates”). The observations in the Cochrane review indicate that screening the whole population for LGA and offering earlier induction of labor to screen positive women could decrease maternal and neonatal morbidity. However, low risk women necessarily have a lower prior risk of complications and in many cases where the baby is thought to be LGA by ultrasound, the result is a false positive. Modeling has demonstrated that very large numbers of low-risk women need to have intervention to prevent relatively small numbers of adverse events (4).

It has been suggested that combining ultrasonic EFW with maternal biochemical markers could improve diagnostic effectiveness (5). We have recently developed a metabolite ratio (see Methods), measured in maternal serum or plasma, which was predictive of fetal growth restriction (FGR) in 2 demographically dissimilar populations. Moreover, we showed that the ratio in combination with EFW could potentially be used as a screening test for FGR to prevent adverse outcome. In this study, we evaluated in nondiabetic women the association between the metabolite ratio and birth weight across the whole range and its ability to predict LGA with the aim of assessing whether the ratio also has potential as a novel screening tool for LGA.

## Methods

The overall approach of the study was (1) to analyze the metabolite ratio and its components measured at 36 wkGA in relation to LGA and birth weight z score at term in the Pregnancy Outcome Prediction (POP) study, (2) to externally validate the POP study results in 24 to 28 wkGA samples from 2 independent subgroups of the Born in Bradford (BiB) study in relation to subsequent LGA and birth weight z score, and (3) to repeat the POP study analysis using measurements from 28 wkGA and the same definitions to allow a direct comparison with the validation results from the BiB study.

### Pregnancy Outcome Prediction Study

The POP study was a prospective cohort study of unselected nulliparous women with a singleton pregnancy attending the Rosie Hospital, Cambridge, UK, between January 2008 and July 2012, and it has been previously described in detail (6,7). In brief, a total of 4512 study participants were recruited at the time of their dating ultrasound and gave written informed consent. Blood samples were collected at the recruitment visit and at further research visits at 20, 28, and 36 wkGA. A total of 4212 women were followed from recruitment through to delivery, after which outcome data were obtained by individual review of patient records and by linkage to the hospital’s electronic records. Gestational diabetes was defined using the criteria adapted from the World Health Organization (2008-2010) or the criteria adapted from the International Association of Diabetes and Pregnancy Study Groups (2011 onward) as previously described (8). Ethical approval was obtained from the Cambridgeshire 2 Research Ethics Committee (reference no. 07/H0308/163).

We previously analyzed serial maternal serum samples (12, 20, 28, and 36 wkGA) using a case cohort analysis in the POP study (9-11); however, the case definition did not include LGA. The details of that study are published elsewhere (9). In brief, we performed metabolomics (see Biochemical Analyses) on maternal serum at 12, 20, and 28 wkGA to identify metabolites predictive of FGR at term (175 cases and 299 controls). We identified 22 predictive metabolites in an internal validation step using 36 wkGA samples from the same women. Nine of these were independently predictive, including 5 that improved the prediction over an established biomarker, the soluble fms-like tyrosine kinase 1:placental growth factor ratio. A tobacco metabolite cotinine N-oxide was excluded from further analysis, and the ratio was based on the remaining 4 metabolites. Our analysis demonstrated that a ratio of the products of the relative concentrations of 2 positively associated metabolites to the product of the relative concentrations of 2 negatively associated metabolites was strongly predictive of FGR at term (see Exposures and Outcomes). In the present study, we sought to determine in nondiabetic women from the random sample (1) whether the metabolite ratio was associated with birth weight across the whole range and (2) whether it was also predictive of LGA.

### External Validation: the Born in Bradford Study

We wrote a prospective analysis plan for external validation of the metabolite ratio and its components at 24 to 28 wkGA in relation to subsequent delivery of LGA infant and birth weight as a continuous z score in the BiB study [Supplemental Panel 1 (12)]. The BiB study, which was conducted between 2007 and 2011, has been described previously in detail (9,13,14). In brief, 12 500 pregnant women of mixed parity and including multiple pregnancies were recruited to the study. All study participants gave written informed consent and ethical approval was obtained from the Bradford Research Ethics Committee. Participation involved collection of maternal data and conduct of an oral glucose tolerance test (OGTT) at 24 to 28 wkGA, excluding women who had already been diagnosed with diabetes (type 1 or 2 or gestational diabetes diagnosed in early pregnancy). The OGTT included storage of fasting maternal plasma for the purposes of research, which was available for 85% of the invited participants (there were no further research blood samples obtained later in pregnancy from BiB participants). From this group, 1000 women (subgroup 1) who had stored fasting plasma and were either white British or Pakistani origin were selected randomly for metabolomics (15). Gestational diabetes was defined using the modified World Health Organization criteria as previously described (16). After the exclusion of 91 women who had gestational diabetes diagnosed on the basis of the OGTT, 909 eligible women were included in this group. Metabolomics was also performed on the 24 to 28 wkGA fasting plasma sample from a second randomly sampled group of 1592 BiB participants (subgroup 2), which did not overlap with the first. Subgroup 2 were selected using a case cohort design with the 1592 eligible for this study (including 1199 healthy women and 393 women belonging to at least 1 of the 6 case groups) reflecting a random cohort sample (15). We excluded 135 women with gestational diabetes leaving 1457 eligible women in the second subgroup. Subgroup 2 was also half white British and half Pakistani origin as these are the main homogeneous ethnic groups in the BiB study. When the 2 BiB study subgroups were pooled, there were 86 cases of LGA and 2280 controls. The data were analyzed both as a single pooled group and in the 2 separate subgroups [Supplemental Panel 1 (12)]. Fully customized birth weight percentiles were not available for all women in the BiB cohort. Therefore, a modified definition of LGA was employed: birth weight >90th percentile corrected for GA and fetal sex following Hadlock (17) [Supplemental Panel 1 (12)].

### Comparing POP and BiB Study Analyses

The primary analysis of the POP study used the 36 wkGA serum sample, as the focus of the study was predicting large babies at term, and 36 wkGA was the latest gestational sampling point in the POP study. The BiB study only had a single gestational time point of blood sampling, which was at 24 to 28 wkGA. Hence, we repeated the POP study analysis using the 28 wkGA metabolite ratio to compare associations between the two cohorts. When we compared data from the POP and BiB studies, the same modified definition of LGA was employed in the POP study to allow a direct comparison between the cohorts (see following discussion).

### Biochemical Analyses

In the POP study, nonfasting maternal serum samples were analyzed, whereas in the BiB study, fasting plasma samples were analyzed. Metabolomic measurements were performed by Metabolon (Morrisville, NC, USA) using ultrahigh performance liquid chromatography-tandem mass spectroscopy as the platform. The details have been described previously (9,18). In brief, a total of 837 metabolites of known identity were quantified. The spectroscopic peak was quantified as the area under the curve for the given metabolite in the given sample. The concentration of the metabolite in a given sample was then calculated as the ratio of the peak from that sample to the median area under the curve of that metabolite peak obtained from all of the assays performed on the given day. Hence, all metabolite concentrations were calculated as relative concentrations, expressed as multiples of the median.

### Exposures and Outcomes

The 4-metabolite ratio [(A × B)/(C × D)], its numerator and denominator, and the 4 individual metabolites were used as exposures in the present analysis. The 4 individual metabolites were (A) 1-(1-enyl-stearoyl)-2-oleoyl-GPC (P-18:0/18:1), (B) 1,5-anhydroglucitol (1,5-AG), (C) 5alpha-androstan-3alpha,17alpha-diol disulfate, and (D) N1,N12-diacetylspermine. The main outcomes were (1) birth weight >90th percentile (LGA) and (2) birth weight z score for gestational age and fetal sex using the UK 1990 reference (19). These outcomes were used in the analysis of the 36 wkGA samples in relation to term outcomes in the POP study. Additionally, birth weight z score, percentile and LGA were defined using the Hadlock formula applied to sex-adjusted birth weights (17) [Supplemental Panel 1 (12)]. These definitions were used in the analysis of the 24 to 28 wkGA samples in relation to any subsequent delivery of LGA infant in the BiB study (external validation) and the analysis of the 28 wkGA samples in the POP study.

### Statistical Methods

In the POP study, z scores for each exposure were calculated from log-transformed values, referent to the random subcohort. The 2 BiB subgroups were independent of each other and analyzed in 2 separate batches; hence, z scores of the log-transformed ratio were calculated separately in the 2 subgroups, and the z scores from the 2 subgroups were subsequently pooled when the BiB data were analyzed as a single group. For each z score transformed exposure and the birth weight z score, a linear regression model was fitted, and the results were reported as the beta coefficient with 95% CI. The birth weight z score was also plotted against the metabolite ratio z score. In the POP study, the analyses were repeated adjusting for maternal age, height, body mass index, smoking status, marital status, ethnicity, and age at discontinuation of full-time education. Discrimination between the LGA cases and noncases was performed in both studies by measuring the area under the receiver operating characteristic curve (AUROCC) and the 95% CI. The metabolite ratio was originally developed to be positively associated with FGR. Consequently, the direction of association with LGA should be in the opposite direction; hence, the inverse of the ratio was used to calculate the AUROCC. The associations between each exposure and LGA were also analyzed using logistic regression, and associations were expressed as the odds ratio (OR) and the 95% CI for a 1 SD increase in the given exposure. *P*-values for external validation were 1-sided as validation was directional based on the observed results in the POP study; that is, we would not regard an association as validated if the *P*-value was below the given threshold but the association was in the opposite direction to that predicted. The metabolite ratio was considered as the main exposure whereas the denominator and numerator of the ratio and the 4 individual metabolites were considered as secondary exposures, and corrections for multiple testing were applied accordingly [Supplemental Panel 1 (12)]. Statistical analysis was performed using Stata version 15.1 and R version 3.4.4.

## Results

### Description of the Random Sample of the POP Study Cohort and Power Calculation

The random sample of the POP study cohort included 313 participants. Of these, 11 had preexisting or gestational diabetes and 21 did not have the metabolite measurements available at 36 wkGA, leaving 281 eligible participants. This group included 15 LGA cases and 266 noncases [Supplemental Figure 1 (12)]. With this sample size, we had a 96% statistical power to identify an equally strong association between the metabolite ratio and LGA to the one previously identified between the metabolite ratio and FGR (AUROCC = 0.78, OR = 2.93), assuming alpha = 0.05 and a 2-sided test. Maternal characteristics including anthropometrics, lifestyle factors, socioeconomic status (20,21) and diseases, and birth outcomes in the LGA cases and noncases are described in Table 1. A comparison between the 281 participants and the remainder of the POP study cohort (n = 3931) is presented in Supplemental Table 1 (12). The participants were representative of the whole cohort in terms of maternal characteristics. Some of these were self-reported. However, the Metabolon assay included cotinine N-oxide, which provides objective evidence of cigarette use. Analyzing the data on self-reported smoking status at 20 weeks with maternal serum cotinine N-oxide levels at 20 weeks [available for 268/281 (95%) of the women] as the gold standard, self-report had a sensitivity of 73% and a specificity of 99% (AUROCC = 0.86). Due to exclusion of preterm births, the gestational age and birth weight distribution differed between the participants and the rest of the cohort but the birth weight z score distributions did not [Supplemental Table 1 (12)].

**Table 1.** Characteristics of the women from the Pregnancy Outcome Prediction study by large for gestational age status

Characteristic	LGA at term (n = 15)	Not LGA at term (n = 266)
Maternal characteristics		
Age, years	30 (28 to 32)	30 (26 to 33)
Age stopped full time education ≥21 years	11 (73)	146 (55)
Missing	0 (0)	1 (<1)
Height, cm	169 (159 to 172)	165 (161 to 169)
Body mass index, kg/m^2^	24 (22 to 26)	24 (22 to 28)
Smoker	0 (0)	11 (4)
Any alcohol consumption	0 (0)	10 (4)
Deprivation, score	10.5 (3.9 to 14.4)	8.5 (5.9 to 13.9)
Deprivation, rank	23182 (18729 to 31041)	25727 (19307 to 28896)
Deprivation rank quintile		
1 (most deprived)	0 (0)	0 (0)
2	1 (7)	17 (6)
3	4 (27)	48 (18)
4	4 (27)	68 (26)
5 (least deprived)	6 (40)	123 (46)
Missing	0 (0)	10 (4)
White ethnicity	15 (100)	250 (94)
Missing	0 (0)	5 (2)
Married	12 (80)	190 (71)
Essential hypertension	0 (0)	5 (2)
Preexisting renal disease	0 (0)	1 (<1)
Preeclampsia	1 (7)	12 (5)
Birth outcomes		
Birth weight, g	4410 (4020 to 4580)	3440 (3135 to 3740)
Birth weight z score, UK 1990	1.42 (1.34 to 1.65)	−0.16 (−0.84 to 0.34)
Birth weight percentile, UK 1990	92 (91 to 95)	44 (20 to 63)
Birth weight percentile category, UK 1990		
<10th	0 (0)	34 (13)
10th to <50th	0 (0)	119 (45)
50th to 90th	0 (0)	113 (42)
>90th	15 (100)	0 (0)
Gestational age, weeks	40.3 (39.9 to 41.3)	40.4 (39.4 to 41.3)
Female fetal sex	6 (40)	135 (51)
Induction of labor	6 (40)	87 (33)
Mode of delivery		
Spontaneous vaginal	6 (40)	139 (52)
Assisted vaginal	3 (20)	59 (22)
Intrapartum caesarean	5 (33)	45 (17)
Prelabor cesarean	1 (7)	21 (8)
Missing	0 (0)	2 (1)

In total, 4212 women completed the POP study. The table describes the population of nondiabetic women from the random subcohort who delivered at term and had metabolite data from the 36 weeks of gestational age (wkGA) visit (n = 281). The flow diagram [Supplemental Figure 1 (12)] describes the selection of term large for gestational age (LGA) cases and controls. Data are expressed as median (interquartile range) or n (%) as appropriate. For variables where there is no category labeled “missing,” data were 100% complete. Maternal age was defined as age at recruitment. All other maternal characteristics were defined by self-report at the 20 wkGA questionnaire, from examination of the clinical case record, or linkage to the hospital’s electronic databases. The weight measurement used in the calculation of body mass index was made at the 12 wkGA visit. Socioeconomic status was quantified using the Index of Multiple Deprivation 2007, which is based on census data from the area of the mother’s postcode (20). Deprivation score is the combined sum of the weighted, exponentially transformed domain rank of the domain score, and higher values indicate more deprivation. Conversely, the most deprived area has the lowest rank (=1) and the least deprived area has the highest rank (=32 482). A national reference distribution from 2010 has been used to analyses the rank in quintiles (1 = most deprived, 5 = least deprived), enabling a comparison with the Born in Bradford study (21). Preeclampsia was defined on the basis of the 2013 American College of Obstetricians and Gynecologists criteria. LGA at term was defined as delivery at ≥37 wkGA and birth weight percentile >90th using the UK 1990 population reference.

### Analysis of the 36 wkGA Samples in the POP Study Cohort

Despite the small numbers, all 4 metabolites at 36 wkGA were associated with the risk of delivering an LGA infant (Table 2). As anticipated, all 4 associations were in the opposite direction to that seen for FGR. Hence, the inverse of the metabolite ratio was also associated with LGA, and interestingly, the strength of association was actually greater than the association originally described for FGR (AUROCC = 0.82, 95% CI 0.73 to 0.91 for LGA, compared with 0.78, 95% CI 0.73 to 0.82 for FGR) (9). All 4 of the metabolites were also associated with the continuous birth weight z score, and the metabolite ratio was more strongly associated with it than any of the individual metabolites [Table 2; Supplemental Figure 2 (12)]. Again, as expected, all associations between metabolite levels and birth weight z score were in the opposite direction compared to FGR. All the associations were similar or slightly stronger after the adjustment for maternal characteristics (Table 2). We did not observe notable differences in the associations by fetal sex. An interaction test *P*-value between the ratio measured at 36 wkGA and fetal sex was 0.20 when the outcome was LGA and 0.66 when the outcome was birth weight z score.

**Table 2.** Maternal serum measurements of metabolites at 36 weeks gestational age in relation to large for gestational age and birth weight z score at term

	LGA (n = 15 cases)		BW z score (n = 281)	
Metabolite	Unadjusted	Adjusted *P*-value	Unadjusted	Adjusted
(A)	0.74 (0.63 to 0.84) 0.012	6.7 × 10^−3^	−0.12 (−0.22 to −0.01) 0.025	−0.11 (−0.21 to −0.01) 0.034
(B)	0.71 (0.59 to 0.83) 0.020	0.028	−0.19 (−0.29 to −0.08) 7.7 × 10^−4^	−0.19 (−0.29 to −0.08) 4.6 × 10^−4^
(C)	0.68 (0.55 to 0.80) 0.062	0.028	0.16 (0.06 to 0.26) 2.6 × 10^−3^	0.21 (0.12 to 0.31) 2.0 × 10^−5^
(D)	0.69 (0.57 to 0.81) 0.034	0.021	0.16 (0.06 to 0.26) 2.1 × 10^−3^	0.18 (0.09 to 0.28) 2.4 × 10^−4^
(A × B)/(C × D)	0.82 (0.73 to 0.91) 6.7 × 10^−5^	2.2 × 10^−5^	−0.29 (−0.39 to −0.19) 2.1 × 10^−8^	−0.33 (−0.42 to −0.23) 2.3 × 10^−11^
(A × B)	0.77 (0.66 to 0.88) 1.3 × 10^−3^	1.5 × 10^−3^	−0.22 (−0.32 to −0.11) 7.8 × 10^−5^	−0.21 (−0.31 to −0.11) 6.5 × 10^−5^
(C × D)	0.73 (0.61 to 0.85) 0.014	5.1 × 10^−3^	0.20 (0.10 to 0.30) 1.6 × 10^−4^	0.25 (0.15 to 0.34) 6.6 × 10^−7^

The data are expressed as the area under the receiver operating characteristic curve (AUROCC) (95% CI) and *P*-value for large for gestational age (LGA), and beta (95% CI) and *P* value for birth weight (BW) z score. The analyses were performed without and with adjustment for maternal age, height, body mass index, smoking status, marital status, ethnicity and age at discontinuation of full time education. AUROCC (95% CI) is given for the unadjusted analysis only. The metabolite ratio [(A × B)/(C × D)] developed to predict fetal growth restriction was calculated from 4 metabolites measured at 36 weeks of gestational age (wkGA): (A) 1-(1-enyl-stearoyl)-2-oleoyl-GPC (P-18:0/18:1); (B) 1,5-anhydroglucitol (1,5-AG); (C) 5alpha-androstan-3alpha,17alpha-diol disulfate; and (D) N1,N12-diacetylspermine. The analysis was restricted to nondiabetic women from the random subcohort who delivered at term and had metabolite data from the 36 wkGA visit (n = 281). BW z scores and percentiles were calculated using the UK 1990 population reference and LGA was defined as BW>90^th^ percentile. AUROCC was based on the metabolite, metabolite ratio or product alone. The *P*-value (2-sided) was calculated from linear regression for log-transformed z scores of metabolites, their ratios and products using Wald test, with the null hypothesis that the coefficient for LGA = 0.

### External Validation Using the 28 wkGA Samples From the BiB Study Cohort

**Figure 1.**
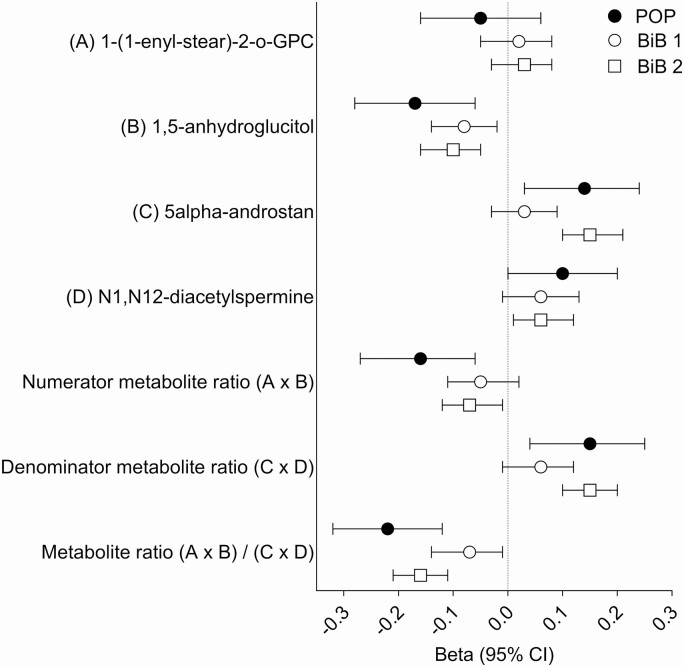
Beta coefficients from regression analyses (95% CI) for metabolite measurements at ~24 to 28 weeks of gestational age (wkGA) and birth weight z score. The estimated beta coefficients are given for 1 SD increase in the log-transformed metabolite product or ratio. The subsequent birth weight z score was corrected only for gestational age and fetal sex. The numbers included in the analysis were 281, 909 and 1457 for the Pregnancy Outcome Prediction (POP) study, Born in Bradford (BiB) subgroup 1 (BiB 1) and BiB subgroup 2 (BiB 2), respectively. Abbreviations: 1-(1-enyl-stear)-2-o-GPC, 1-(1-enyl-stearoyl)-2-oleoyl-GPC (P-18:0/18:1); 5alpha-androstan, 5alpha-androstan-3alpha,17alpha-diol disulfate.

**Figure 2.**
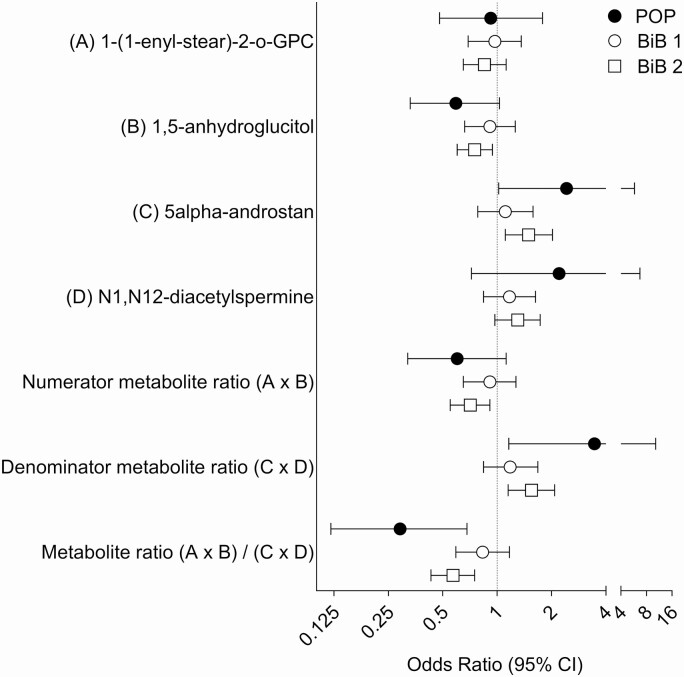
Odds ratios (95% CI) of metabolite measurements at ~24 to 28 weeks of gestational age (wkGA) in relation to large for gestational age (LGA) at birth. Odds ratios are given for 1 SD increase in the log-transformed metabolite, product or ratio. LGA was defined as birth weight >90th percentile, corrected only for gestational age and fetal sex. The Pregnancy Outcome Prediction (POP) study included 9 LGA cases and 286 controls, the Born in Bradford (BiB) subgroup 1 (BiB 1) included 34 LGA cases and 875 controls, and the BiB subgroup 2 (BiB 2) included 52 LGA cases and 1405 controls. Abbreviations: 1-(1-enyl-stear)-2-o-GPC, 1-(1-enyl-stearoyl)-2-oleoyl-GPC (P-18:0/18:1); 5alpha-androstan, 5alpha-androstan-3alpha,17alpha-diol disulfate.

**Figure 3.**
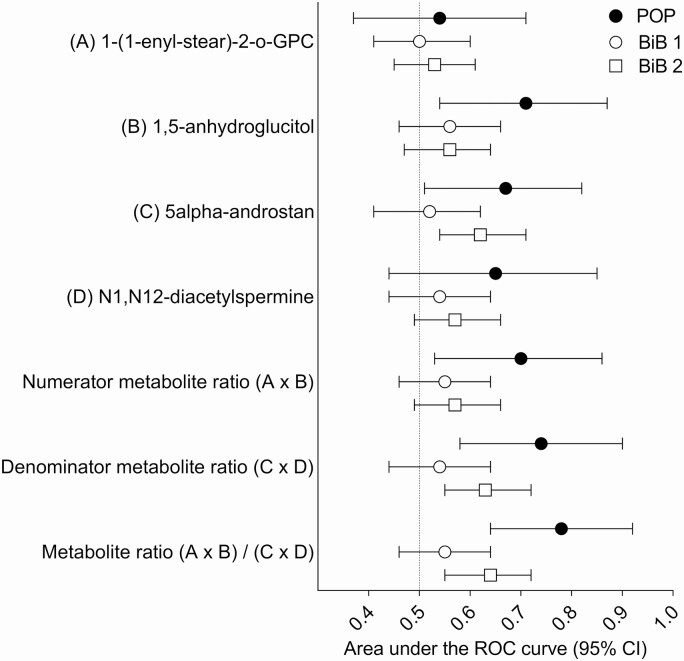
Area under the receiver operating characteristic curve (95% CI) for metabolite measurements at ~24 to 28 weeks of gestational age (wkGA) in relation to large for gestational age (LGA) at birth. LGA was defined as birth weight >90th percentile, corrected only for gestational age and fetal sex. The Pregnancy Outcome Prediction (POP) study included 9 LGA cases and 286 controls, the Born in Bradford (BiB) subgroup 1 (BiB 1) included 34 LGA cases and 875 controls, and the BiB subgroup 2 (BiB 2) included 52 LGA cases and 1405 controls. Abbreviations: 1-(1-enyl-stear)-2-o-GPC, 1-(1-enyl-stearoyl)-2-oleoyl-GPC (P-18:0/18:1); 5alpha-androstan, 5alpha-androstan-3alpha,17alpha-diol disulfate.

The selection and the characteristics of the women from the BiB study are described in Supplemental Figures 3 and 4 and Supplemental Tables 2 and 3 (12). The inverse association between the metabolite ratio and the continuous birth weight z score was validated in the data from the BiB study (beta = −0.12 SD, *P =* 1.3 × 10^−9^). The ratio was also predictive of LGA (AUROCC = 0.60, 95% CI 0.54 to 0.67, *P =* 8.6 × 10^−5^). Interestingly, when the 2 BiB subgroups were analyzed separately, the association with birth weight was weaker in the first sub-group compared with the second (beta = −0.07 and −0.16 SD, respectively) (Figure 1). Similarly, the association between the metabolite ratio at 24 to 28 wkGA and the risk of delivering an LGA infant differed comparing the 2 BiB subgroups. There was no/weak evidence for an association between the ratio and the risk of LGA in BiB sub-group 1 (n = 34 cases and 875 controls, OR = 0.83, 95% CI 0.59 to 1.17, 1-sided *P =* 0.15, AUROCC = 0.55, 95% CI 0.46 to 0.64). However, there was strong evidence for an association between the metabolite ratio and the risk of LGA in BiB subgroup 2 (n = 52 cases and 1405 controls, OR = 0.57, 95% CI 0.43 to 0.75, one-sided *P =* 3 × 10^−5^, AUROCC = 0.64, 95% CI 0.55 to 0.72) (Figures 2 and 3).

### Analysis of the 28 wkGA Samples in the POP Study Cohort

Analysis of 28 wkGA samples in the POP study demonstrated that a 1 SD increase in the log-transformed metabolite ratio was associated with a −0.22 SD (95% CI −0.12 to −0.32) change in birth weight z score. Moreover, using the same modified definition of LGA (to allow direct comparison with BiB), the 28 wkGA measurement of the ratio was also associated with LGA in the POP study (n = 9 cases and 286 controls, AUROCC = 0.78, 95% CI 0.64 to 0.92). Hence, the point estimates for BiB subgroup 2 were within the 95% CI of the POP study values relating to the 28 wkGA blood sample, whereas those of subgroup 1 fell outside this range.

## Discussion

The main finding of the present study was that a metabolite ratio, which we had previously described as a predictor for FGR (9), was also associated with birth weight across the whole range and was similarly predictive for the risk of delivering an LGA infant as it was for predicting FGR. We identified these associations in a random sample of participants from the POP study who had been used as a comparison group in the previous analysis of FGR. As the current hypothesis tests were independent of the previous analysis, the findings are unlikely to be data-driven and, given the very low *P*-values, are also unlikely to have occurred by the play of chance. The associations with both birth weight z score and the risk of delivering an LGA infant were also validated in a separate group of cases and controls from the BiB study, another pregnancy cohort. Validation was observed despite the fact that the BiB cohort was highly dissimilar from the POP study (a very deprived population, mixed Pakistani and white British heritage, and mixed parity) and employed different sample types (fasting plasma vs nonfasting serum). We conclude that there is strong evidence to indicate that the metabolite ratio is predictive of fetal growth across the whole range of fetal size. Hence, it may offer clinically useful prediction of LGA, as well as of FGR.

The clinical importance of the current work is that this test has potential for use as a screening test in combination with ultrasound. Screening all pregnant women with ultrasound is not currently recommended due to lack of direct evidence that it is clinically and economically effective (22). One of the explanations for the lack of a beneficial effect may be that many infants born to women who are suspected to have an LGA fetus are born with birth weight in the normal range. Moreover, there is evidence that women with a false-positive diagnosis of fetal LGA are at increased risk of unnecessary intervention (23-25). Hence, the benefit yielded by screening and intervention in true positives may be outweighed by iatrogenic harm in false positives. Previously, we demonstrated that the combination of the metabolite ratio and ultrasonic EFW had a higher positive predictive value in the prediction of FGR for a given proportion of screen positives compared to the combination of ultrasonic EFW and soluble fms-like tyrosine kinase 1:placental growth factor ratio. Determining whether the combination of ultrasonic suspicion of LGA and measurement of the metabolite ratio provides enhanced prediction of macrosomia over ultrasound on its own is an important area for future study.

The biological plausibility of associations between each of the metabolites and fetal growth has previously been discussed (9). However, 2 of the associations are particularly relevant in the context of the endocrinology of pregnancy. The control of glucose in pregnancy is complex with a state of insulin resistance combined with stimulation of insulin production by lactogenic hormones (26). 1,5-AG is inversely associated with blood glucose, and we have previously shown that 1,5-AG levels fall with advancing gestational age (9). The inverse association between 1,5-AG and birth weight suggests that the decline in 1,5-AG is greater with increased fetal size, and this could reflect failure of the control of euglycemia in the face of insulin resistance but without overt gestational diabetes. Similarly, we have previously shown that 5alpha-androstan-3alpha,17alpha-diol disulfate was at very low levels in maternal serum in the first trimester and levels massively increase with advancing gestational age (9). It is likely that this steroid is part of the fetal-placental-maternal unit of production of estrogens (27). Hence, it is plausible that higher levels could reflect greater fetal or placental synthetic capacity. However, in both cases, the mechanistic details remain to be determined.

A relative weakness of the present study is that the gestational age of collection of blood in BiB was at a much earlier stage in pregnancy (24-28 wkGA) than that for the main analyses in the POP study (36 wkGA). We addressed this by comparing the associations with the POP study sample collected at 28 wkGA. However, we anticipate that the ultimate clinical application relating to screening for LGA will take place near term, as the main intervention is earlier term delivery. Ideally, external validation would also have involved analysis of samples at around 36 wkGA. Analysis of the BiB data confirmed the presence of associations with both birth weight z score and with the risk of delivering an LGA infant. The associations were generally weaker, but external validation results are often weaker than those found in the discovery sample (a phenomenon known as “winner’s curse”). However, a further complexity is that the strength of association differed comparing the 2 subgroups of the BiB study. This inconsistency is difficult to explain, given the similarity between the 2 subgroups and given that we observed consistent associations between the 2 subgroups when we analyzed FGR. It may be explained partly by the method for quantifying metabolites in the samples. The metabolomics platform employed in both studies was a discovery platform and the method did not include isotopically labeled standard but rather calculated relative concentrations. More consistent results might be achieved when all metabolites are quantified by the actual molar concentration, and this is an area for future study, as well as being a necessary step for clinical development of the assay.

Among the women included in the analysis from the POP study (n = 281), the proportion of LGA was about 5%, and the number of women delivering an LGA infant was low (n = 15). This was partly due to the deliberate exclusion of diabetic women and partly because nulliparous women deliver smaller infants on average. For example, with these numbers, estimation of the association between 5alpha-androstan-3alpha,17alpha-diol disulfate and LGA resulted in a *P*-value of 0.062 but an AUROCC of 0.68. The likelihood that these values are consistent with a true association with LGA is supported by the observation that the metabolite was strongly associated with the birth weight z score. We overcame the limitation of small numbers by choosing a larger cohort with a mixed parity for validation. The total number of LGA infants included in the analysis from the BiB study was 86 (the proportion of LGA was 4%), and therefore there was much less uncertainty in the estimation of the associations between LGA and the metabolites. The potential impact of fasting on the metabolite ratio could not be directly assessed in the present analysis, and this could be an area for future study. Another interesting area for further study would be to examine the association between the ratio and birth weight related long-term childhood health conditions.

Although our validation cohort consisted of ~50% women of Pakistani ethnicity, there were small proportions of other ethnic groups such as black Africans in both POP and BiB study cohorts. Therefore, further external validation is needed to assess the generalizability of our results in other ethnic groups.

The analyses adjusted for maternal characteristics in the POP study demonstrated that the observed associations were robust. Moreover, self-reported smoking had a high sensitivity and very high specificity against maternal serum cotinine N-oxide levels. Hence, we do not believe that the results are likely to be affected by the self-report of maternal variables.

We were not able to perform an analysis of fully customized birth weight percentiles in the BiB study. However, the utility of customization is not universally accepted. For example, 1 of the largest studies in fetal growth in the last 10 years (InterGrowth 21st) does not support customization (28), and there is no international consensus about its benefit. We have previously shown in the POP study that the independent association between the birthweight percentile and adverse pregnancy outcome is similar regardless of customization (29). Furthermore, previous work has also shown that partial customization made birthweight a poorer predictor of adverse outcome at term (30).

To conclude, we have shown than a recently described metabolite ratio which is predictive for FGR is also associated with birth weight across the whole range, including LGA. The ratio has the potential to improve the prediction of LGA over the current methods. Further studies using a quantitative version of the assay and tested in late pregnancy in other populations would strengthen the case for the ratio’s potential clinical utility in this context.

## Data Availability

Restrictions apply to the availability of data generated or analyzed during this study to preserve patient confidentiality or because they were used under license. The corresponding authors will on request detail the restrictions and any conditions under which access to some data may be provided. Data requests on the POP study data can be made to U.S. or G.C.S.S. and data requests on the BiB study data can be made to D.A.L.

## References

[1] Henriksen T . The macrosomic fetus: a challenge in current obstetrics. Acta Obstet Gynecol Scand. 2008;87(2):134-145. 18231880 10.1080/00016340801899289

[2] Sebert S , Lowry E, Aumuller N, et al. Cohort Profile: The DynaHEALTH consortium—a European consortium for a life-course bio-psychosocial model of healthy ageing of glucose homeostasis. Int J Epidemiol. 2019;48(4):1051-1051k. 31321419 10.1093/ije/dyz056 PMC6693805

[3] Boulvain M , Irion O, Dowswell T, Thornton JG. Induction of labour at or near term for suspected fetal macrosomia. Cochrane Database Syst Rev. 2016(5):CD000938. 10.1002/14651858.CD000938.pub2 PMC7032677 27208913

[4] Rouse DJ , Owen J, Goldenberg RL, Cliver SP. The effectiveness and costs of elective cesarean delivery for fetal macrosomia diagnosed by ultrasound. JAMA. 1996;276(18):1480-1486. 8903259

[5] Araujo Júnior E , Peixoto AB, Zamarian AC, Elito Júnior J, Tonni G. Macrosomia. Best Pract Res Clin Obstet Gynaecol. 2017;38:83-96. 27727018 10.1016/j.bpobgyn.2016.08.003

[6] Gaccioli F , Lager S, Sovio U, Charnock-Jones DS, Smith GCS. The pregnancy outcome prediction (POP) study: Investigating the relationship between serial prenatal ultrasonography, biomarkers, placental phenotype and adverse pregnancy outcomes. Placenta. 2017;59(suppl 1):17-25.

[7] Sovio U , White IR, Dacey A, Pasupathy D, Smith GCS. Screening for fetal growth restriction with universal third trimester ultrasonography in nulliparous women in the Pregnancy Outcome Prediction (POP) study: a prospective cohort study. Lancet. 2015;386(10008):2089-2097. 26360240 10.1016/S0140-6736(15)00131-2 PMC4655320

[8] Sovio U , Murphy HR, Smith GC. Accelerated fetal growth prior to diagnosis of gestational diabetes mellitus: a prospective cohort study of nulliparous women. Diabetes Care. 2016;39(6):982-987. 27208333 10.2337/dc16-0160

[9] Sovio U , Goulding N, McBride N, et al. A maternal serum metabolite ratio predicts fetal growth restriction at term. Nat Med. 2020;26(3):348-353. 32161413 10.1038/s41591-020-0804-9

[10] Jayasuriya NA , Hughes AE, Sovio U, Cook E, Charnock-Jones DS, Smith GCS. A lower maternal cortisol-to-cortisone ratio precedes clinical diagnosis of preterm and term preeclampsia by many weeks. J Clin Endocrinol Metab. 2019;104(6):2355-2366. 30768664 10.1210/jc.2018-02312 PMC6500797

[11] Gong S , Sovio U, Aye IL, et al. Placental polyamine metabolism differs by fetal sex, fetal growth restriction, and preeclampsia. JCI insight. 2018;3(13):e120723. 10.1172/jci.insight.120723 PMC6124516 29997303

[12] Sovio U , Goulding N, McBride N, et al. Data from: A maternal serum metabolite ratio predicts large for gestational age infants at term: a prospective cohort study. Apollo: University of Cambridge Repository. Deposited October 15, 2021. 10.17863/CAM.76865 PMC8947792 34897472

[13] Raynor P ; Born in Bradford Collaborative Group. Born in Bradford, a cohort study of babies born in Bradford, and their parents: protocol for the recruitment phase. BMC Public Health. 2008;8:327. 18811926 10.1186/1471-2458-8-327 PMC2562385

[14] Wright J , Small N, Raynor P, et al; Born in Bradford Scientific Collaborators Group. Cohort profile: the Born in Bradford multi-ethnic family cohort study. Int J Epidemiol. 2013;42(4):978-991. 23064411 10.1093/ije/dys112

[15] Taylor K , McBride N, Goulding NJ, et al. Metabolomics datasets in the Born in Bradford cohort. Wellcome Open Research. 2020;5:264. 10.12688/wellcomeopenres.16341.2 PMC11109709 38778888

[16] West J , Santorelli G, Whincup PH, et al. Association of maternal exposures with adiposity at age 4/5 years in white British and Pakistani children: findings from the Born in Bradford study. Diabetologia. 2018;61(1):242-252. 29064033 10.1007/s00125-017-4457-2 PMC6046463

[17] Hadlock FP , Harrist RB, Martinez-Poyer J. In utero analysis of fetal growth: a sonographic weight standard. Radiology. 1991;181(1):129-133. 1887021 10.1148/radiology.181.1.1887021

[18] Sovio U , McBride N, Wood AM, et al. 4-Hydroxyglutamate is a novel predictor of pre-eclampsia. Int J Epidemiol. 2020;49(1):301-311. 31098639 10.1093/ije/dyz098 PMC7124498

[19] Freeman JV , Cole TJ, Chinn S, Jones PR, White EM, Preece MA. Cross sectional stature and weight reference curves for the UK, 1990. Arch Dis Child. 1995;73(1):17-24. 7639543 10.1136/adc.73.1.17 PMC1511167

[20] Noble M , McLennan D, Wilkinson K, Whitworth A, Barnes H, Dibben C. The English Indices of Deprivation 2007. Department for Communities and Local Government (UK); 2008.

[21] McLennan D , Barnes H, Noble M, Davies J, Garratt E, Dibben C. The English Indices of Deprivation 2010. Department for Communities and Local Government (UK); 2011.

[22] Wastlund D , Moraitis AA, Thornton JG, et al. The cost-effectiveness of universal late-pregnancy screening for macrosomia in nulliparous women: a decision analysis. BJOG. 2019;126(10):1243-1250. 31066982 10.1111/1471-0528.15809 PMC6771727

[23] Little SE , Edlow AG, Thomas AM, Smith NA. Estimated fetal weight by ultrasound: a modifiable risk factor for cesarean delivery? Am J Obstet Gynecol. 2012;207(4):309.e1-309.e6. 22902073 10.1016/j.ajog.2012.06.065

[24] Blackwell SC , Refuerzo J, Chadha R, Carreno CA. Overestimation of fetal weight by ultrasound: does it influence the likelihood of cesarean delivery for labor arrest? Am J Obstet Gynecol. 2009;200(3):340.e1-340.e3. 19254597 10.1016/j.ajog.2008.12.043

[25] Parry S , Severs CP, Sehdev HM, Macones GA, White LM, Morgan MA. Ultrasonographic prediction of fetal macrosomia. Association with cesarean delivery. J Reprod Med. 2000;45(1):17-22. 10664942

[26] Vejrazkova D , Vcelak J, Vankova M, et al. Steroids and insulin resistance in pregnancy. J Steroid Biochem Mol Biol. 2014;139:122-129. 23202146 10.1016/j.jsbmb.2012.11.007

[27] Berkane N , Liere P, Oudinet JP, et al. From pregnancy to preeclampsia: a key role for estrogens. Endocr Rev. 2017;38(2):123-144. 28323944 10.1210/er.2016-1065

[28] Villar J , Cheikh Ismail L, Victora CG, et al; International Fetal and Newborn Growth Consortium for the 21st Century (INTERGROWTH-21st). International standards for newborn weight, length, and head circumference by gestational age and sex: the Newborn Cross-Sectional Study of the INTERGROWTH-21st Project. Lancet. 2014;384(9946):857-868. 25209487 10.1016/S0140-6736(14)60932-6

[29] Sovio U , Smith GCS. The effect of customization and use of a fetal growth standard on the association between birthweight percentile and adverse perinatal outcome. Am J Obstet Gynecol. 2018;218(2S):S738-S744. 29199029 10.1016/j.ajog.2017.11.563

[30] Iliodromiti S , Mackay DF, Smith GC, et al. Customised and noncustomised birth weight centiles and prediction of stillbirth and infant mortality and morbidity: a cohort study of 979,912 term singleton pregnancies in Scotland. PLoS Med. 2017;14(1):e1002228. 28141865 10.1371/journal.pmed.1002228 PMC5283655

