# Erratum to: A Maternal Serum Metabolite Ratio Predicts Large for Gestational Age Infants at Term: A Prospective Cohort Study

**DOI:** 10.1210/clinem/dgac036

**Published:** 2022-02-02

In the above-named article by Sovio U, Goulding N, McBride N, Cook E, Gaccioli F, Charnock-Jones DS, Lawlor DA, and Smith GCS (*J Clin Endo Metab.* 2021; doi: 10.1210/clinem/dgab842), the following error occurred in Table 2:

The fourth column heading “Adjusted *P*-value” should read “Adjusted.”

The publisher acknowledges the error.

The manuscript has been corrected online.

doi: 10.1210/clinem/dgab842

